# CTCFL regulates the PI3K-Akt pathway and it is a target for personalized ovarian cancer therapy

**DOI:** 10.1038/s41540-022-00214-z

**Published:** 2022-02-07

**Authors:** Marisol Salgado-Albarrán, Julian Späth, Rodrigo González-Barrios, Jan Baumbach, Ernesto Soto-Reyes

**Affiliations:** 1 Departamento de Ciencias Naturales, Universidad Autónoma Metropolitana-Cuajimalpa (UAM-C), Mexico City, Mexico; 2 Chair of Experimental Bioinformatics, TUM School of Life Sciences Weihenstephan, Technical University of Munich, Munich, Germany; 3 Chair of Computational Systems Biology, University of Hamburg, Hamburg, Germany; 4 Unidad de Investigación Biomédica en Cáncer, Instituto Nacional de Cancerología, Mexico City, Mexico; 5 Computational BioMedicine lab, Institute of Mathematics and Computer Science, University of Southern Denmark, Odense, Denmark

**Keywords:** Biomarkers, Cancer, Computational biology and bioinformatics

## Abstract

High-grade serous ovarian carcinoma (HGSC) is the most lethal gynecologic malignancy due to the lack of reliable biomarkers, effective treatment, and chemoresistance. Improving the diagnosis and the development of targeted therapies is still needed. The molecular pathomechanisms driving HGSC progression are not fully understood though crucial for effective diagnosis and identification of novel targeted therapy options. The oncogene CTCFL (BORIS), the paralog of CTCF, is a transcriptional factor highly expressed in ovarian cancer (but in rarely any other tissue in females) with cancer-specific characteristics and therapeutic potential. In this work, we seek to understand the regulatory functions of CTCFL to unravel new target genes with clinical relevance. We used in vitro models to evaluate the transcriptional changes due to the presence of CTCFL, followed by a selection of gene candidates using *de novo* network enrichment analysis. The resulting mechanistic candidates were further assessed regarding their prognostic potential and druggability. We show that CTCFL-driven genes are involved in cytoplasmic membrane functions; in particular, the PI3K-Akt initiators EGFR1 and VEGFA, as well as ITGB3 and ITGB6 are potential drug targets. Finally, we identified the CTCFL targets *ACTBL2*, *MALT1* and *PCDH7* as mechanistic biomarkers to predict survival in HGSC. Finally, we elucidated the value of CTCFL in combination with its targets as a prognostic marker profile for HGSC progression and as putative drug targets.

## Introduction

High-grade serous carcinoma (HGSC) is the most common type of ovarian cancer (OC) and the most lethal gynecologic malignancy^1^. The main reasons for the high mortality is the late diagnosis due to the lack of reliable biomarkers (60% of the tumors are detected once they have metastasized)^2^ and lack of effective treatment; i.e., current treatment involves surgical resection and standard chemotherapy, which has several side effects and tumors usually become resistant to it^3^. Thus, improving the diagnosis and the development of novel targeted therapies for HGSC is an ongoing research task since few previously proposed targeted therapies have been tested^4^. Furthermore, OC is a highly heterogeneous disease and the molecular mechanisms that drive OC progression and chemoresistance are not fully understood. Therefore, the identification of molecular pathway activity aberration in OC is a crucial first step in the development of effective diagnosis and novel targeted therapy options. Recent genome-wide studies show that, at the genetic level, the most frequent alterations in HGSC are in the P53 pathway, including mutations in the TP53 gene^5^. At the transcriptional level, Immuno, hormone-related, and MAPK signaling pathways are deregulated in specific clusters of HGSC patients^6^.

Furthermore, integrative omic analyses of OC tumors indicate that the protein CTCFL is a relevant molecular driver of OC^7,8^. *CTCFL* (BORIS) is the paralog of the *CTCF* gene, which encodes a ubiquitous well-known transcription factor (TF) with an 11-zinc-finger DNA-binding domain that recognizes binding sites (BSs) in the genome and participates in the establishment of chromatin organization and transcriptional regulation^9,10^. Likewise, CTCFL is a transcriptional regulator that competes for the same BSs with CTCF^11^. CTCFL has gained interest because omics data show that it is a cancer-specific protein: it is highly expressed in OC but absent in healthy normal tissue in women, except in testis (www.proteinatlas.org/ENSG00000124092-CTCFL)^9,12,13^. Due to its particular tissue specificity, CTCFL is classified as a cancer-testis antigen (CTA), a family of proteins with therapeutic importance in cancer^14,15^. Notably, *CTCFL* is considered an oncogene^16^ and appears to be a master TF that maintains a stemness state in cancer^17^ through the transcriptional regulation of several well-characterized oncogenes and also other CTAs in several cancer types^18–21^.

Integrative OC-specific omic analyses have found that CTCFL is a molecular marker of HGSC in three different levels: DNA-methylation, gene expression, and DNA copy number^22^. Also, CTCFL plays an important role in the progression of HGSC; for instance, Hillman *et al*. demonstrated that CTCFL expression could be a key factor of initiation^8^ and additional studies indicate that CTCFL can contribute to OC progression through different mechanisms; such as androgen receptor-associated pathway^7^, dysregulation of hTERT telomerase^23^ and it is associated with poor prognosis and advanced stage^24^.

Together, the above makes CTCFL an exciting and promising unique target for OC treatment or biomarker development^25,26^. Despite the notable improvements in the study of CTCFL in cancer, a detailed examination of the transcriptional effects of CTCFL expression in OC remains to be fully characterized, as well as the extent to which it influences other cancer-related processes and their potential application for the development of mechanistic therapies for OC.

In this work, we study the regulatory functions of CTCFL in OC at the transcriptional level to identify mechanistic targets with clinical relevance in OC. We used in vitro models to evaluate the transcriptional changes due to knockdown and overexpression of *CTCFL*; followed by a selection of gene candidates, the identification of CTCFL-DNA-binding sites and *de novo* pathway enrichment analysis for the identification of potential OC driver mechanisms controlled by CTCFL. Finally, the expression level of candidate genes were utilized as endophenotypic marker profiles to assess their prognostic power as molecular signature and their druggability potential.

## Results

### CTCFL-regulated genes participate in cell motility, membrane transport, and extracellular matrix-related processes

**Fig. 1:**
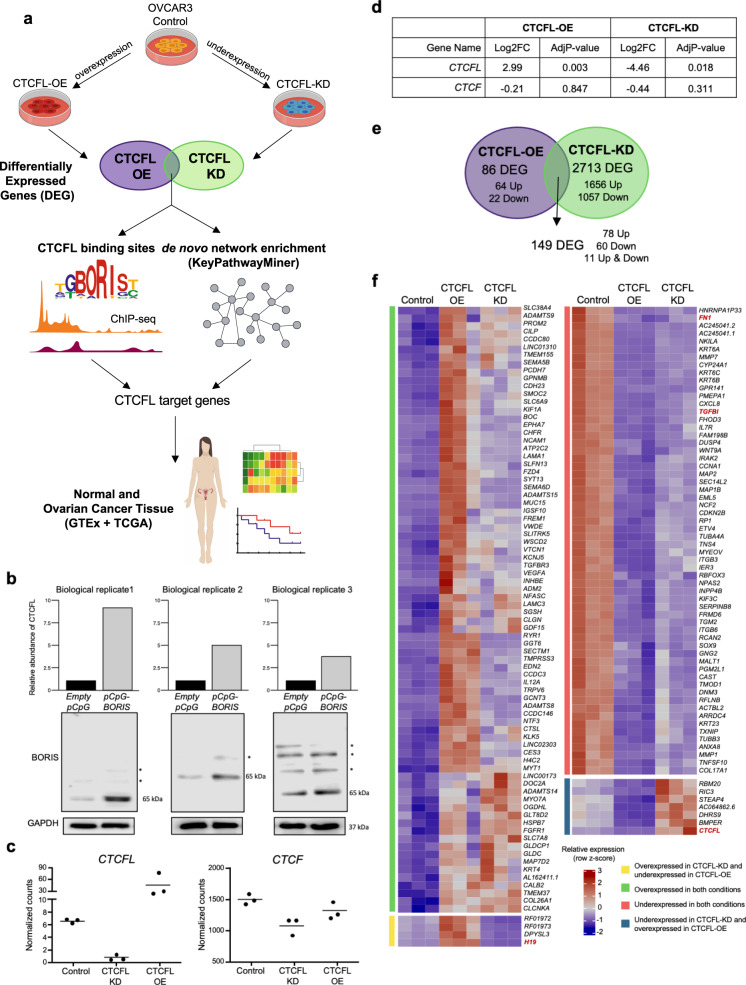
CTCFL-driven DEGs in OVCAR3 cell line. **a** Schematic representation of the workflow followed in this study. RNA-seq data were obtained from OVCAR3 cell lines with *CTCFL* overexpressed and underexpressed from Salgado-Albarran et al.^7^ and subjected to differential expression analysis. Next, CTCFL BS identification and network enrichment analysis was performed to obtain a candidate set of genes. Finally, the candidate gene list was evaluated in OC-derived tumor samples and normal tissue. **b** Experimental validation of CTCFL overexpression at the protein level by Western Blot. The upper panel shows the protein abundance obtained by densitometry. BORIS band is 65 kDa. (*) indicate nonspecific bands. **c** Transcript levels of *CTCFL* and *CTCF* in each experimental condition. **d** Change in gene expression and statistical confidence of *CTCFL* and *CTCF* in each experimental condition vs control. **e** Venn diagram with the number of DEGs found in each and shared between experimental conditions. **f** Relative gene expression (row *z*-score of normalized reads) of the 149 DEGs found in both experimental conditions classified by the direction of gene expression change. Gene names in red indicate previously reported CTCFL transcriptional targets^7,31,37,89^.

In this study, we aim to identify genes regulated by CTCFL that are of clinical importance in OC, either because they are associated with prognosis or because they could be useful as targeted therapy. To address this goal, we used an OC-derived cell line model (OVCAR3) and OC tumor samples (TCGA) to follow three main analysis steps (Fig. 1a). Characterization of *CTCFL* transcript and protein levels was previously published in^7^, showing that it is an HGSOC-derived cell line with high levels of *CTCFL*, making it an appropriate model for the following experiments. First, we evaluated the transcriptional profiles of OVCAR3 cells with knockdown and overexpression of *CTCFL* to identify the differentially expressed genes (DEGs) due to the presence of CTCFL. Following, we selected gene candidates by examining the DEGs with two complementary analyses, the identification of CTCFL binding sites (BSs) and *de novo* pathway enrichment analysis. Finally, the expression level of candidate genes (33 genes + *CTCFL*) was further analyzed in normal (GTEx) and ovarian tumor (TCGA) samples to assess their prognostic and druggable potential.

OC shows the highest expression of *CTCFL* among cancer types; however, the downstream genes and pathways deregulated by it are not fully described in OC. In order to obtain a more detailed landscape of the transcriptional changes occurring due to *CTCFL* expression and identify potential prognostic and druggable targets for treatment, we performed a differential expression analysis with RNA-seq data obtained from two cellular conditions: *CTCFL* knockdown cells (CTCFL-KD) previously characterized^7^ and the overexpression of *CTCFL* (CTCFL-OE). The increase in the protein levels of the experimental conditions was verified by Western Blot (Fig. 1b and Supplementary Fig. 1a) and at the transcript level (Fig. 1c), where *CTCFL* transcripts show a 2.99 log2 fold increase and a 4.46 log2 fold decrease (Fig. 1d) in the corresponding conditions. Furthermore, we also confirm that *CTCF* expression levels did not vary (Fig. 1d); thus, confirming that the experimental approach to modify CTCFL expression was specific.

Once the experimental conditions were validated at the expression and protein levels, two differential expression analyses were performed with the transcriptional data: CTCFL-OE vs Control and CTCFL-KD vs Control. A comparative examination of the identified DEGs shows that 86 DEGs are found when *CTCFL* is overexpressed only, 2713 DEGs when CTCFL is decreased and 149 DEGs are found in both conditions (Fig. 1e). PCA plot of the samples (Supplementary Fig. 1b) shows that the difference in the number of DEGs found in each condition is due to little change in OE compared to KD, and not because of sample variation within a condition. A possible explanation for this is the fact the OVCAR3 is a cancer cell line that already expresses *CTCFL*; thus, the exogenous expression might not have a big effect on transcription because target binding sites are already occupied, while the decrease using KD leads to great differences in expression even with small changes in *CTCFL* levels.

Functional enrichment analysis of the DEG found with the overexpression of *CTCFL* (86 genes) shows that the proteins encoded by them are localized mainly in the cytoplasmic membrane and cell periphery compartment (Supplementary Fig. 1c). Notably, among the deregulated genes localized in these compartments are membrane transporters; such as members of the solute carrier family (*SLC26A7*, *SLC8A1*, *SLC6A12*, *SLC7A2*, and *SLC03A1*) and ATP-binding cassette (*ABCC6*). Likewise, we identified cytoskeleton-related proteins (*SGIP1*, *MAPT*, *PACSIN1*, *ACTG1*, and *VIM*), cell adhesion-related (*SCUBE1*, *ICAM5*, and *BVES*), motility-related (*ENPP2*, *CSPG4*, and *C5AR1*), metalloproteinases (*ADAM11*), the insulin receptor (*INSR*), and one of its known ligands (*IGF1*) (Supplementary Fig. 1d).

Likewise, when evaluating the DEGs after the knockdown of *CTCFL* (2713 genes), components of the cell membrane, cell periphery, and cytoskeleton stand out for being differentially expressed (Supplementary Fig. 2a). In particular, similar to the observed in the opposite experimental condition, 50 members of the solute carrier family are differentially expressed (Supplementary Fig. 2b) and cytoskeleton-related proteins (122 DEGs), including *ACTB* and *ACTR2* (Supplementary Fig. 2c). Together, the latter indicates that, while the majority of the genes affected by the presence and absence of CTCFL are different, at the functional level, many of these genes belong to the same protein families or participate in the same cellular processes; i.e., cell motility (cytoskeleton), and membrane transport, which are well-known mechanisms associated with the development of metastases^27,28^ and tumor resistance to drugs^29,30^; suggesting that CTCFL could play an important role in OC metastases.

After the examination of the DEGs uniquely identified in the experimental conditions, we also explored the DEGs found in both conditions: *CTCFL* overexpression and underexpression (149 genes). In this case, we also found previously validated targets of CTCFL (Supplementary Fig. 3a, b), such as *TGFB1*^31^, *FN1*^7^, and the *H19* gene, which has been widely studied as a target of CTCFL and CTCF given its DNA-methylation dependent transcriptional regulation^32,33^.

In addition to the already-known targets, we further identified relevant cancer-associated genes; such as, *FGFR1*, *VEGFA*, *WNT9A*, and *FZD4* (Fig. 1f). Moreover, we found that similar to the described above, the DEGs are mainly involved in the extracellular matrix or cell motility-related processes, such as actin (*ACTBL2*), kinesins (*KIF1A* and *KIF3C*), laminin (*LAMC3* and *LAMA*), microtubule-associated (*MAP2* and *MAP1B*) and solute carrier family genes (*SLC7A8*, *SLC38A4*, and *SLC6A9*) (Fig. 1f and Supplementary Fig. 3c).

Together, these results show that, in general, the cellular effect due to the presence of CTCFL is similar, being the most relevant effects, those associated with extracellular matrix functions, cell motility, and cytoplasmic membrane transporters. Furthermore, these cellular effects are achieved through diverse deregulated genes between experimental conditions.

### CTCFL-induced protein interaction network is composed of key PI3K-Akt signaling pathway and extracellular matrix genes

After examining the DEGs found in each experimental condition, we aimed to select relevant genes with potential mechanistic molecular importance in OC by analyzing the shared DEGs with two approaches: the identification of CTCFL BSs and *de novo* protein pathway enrichment analysis.

**Fig. 2:**
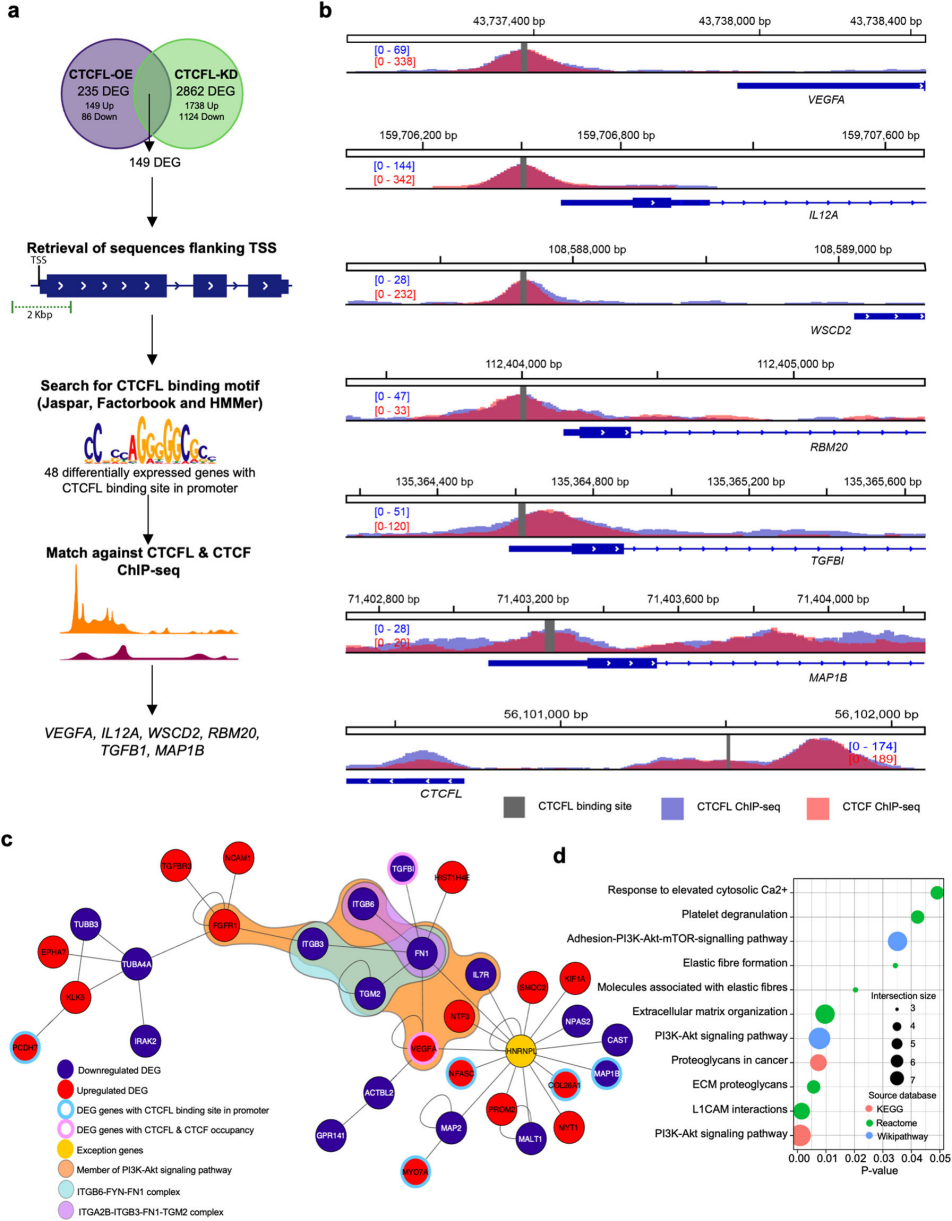
Identification of CTCFL direct targets and CTCFL-deregulated protein interaction network. **a** Schematic representation of the workflow followed for the identification of DEGs with CTCFL BS. Sequences flanking the TSS of DEGs found in both experimental conditions were retrieved and used as input for motif identification with HMMer using the reported CTCFL motifs in Jaspar and Factorbook. Next, genes with a predicted BS and experimentally validated CTCFL and CTCF occupancy (ChIP-seq) were selected. **b** Visual representation of CTCFL and CTCF occupancy in predicted CTCFL BSs of the genes *VEGFA, IL12A, WSCD2, RBM20, TGFB1, MAP1B*, and *CTCFL*. **c** Protein interaction network enriched with CTCFL-deregulated genes found in vitro. **d** Functional enrichment analysis of genes in the protein interaction network using KEGG, Reactome, and Wikipathway databases.

First, we searched BSs in the promoters of the 149 DEGs using the reported DNA motif of CTCFL (Jaspar and Factorbook) and the HMMer tools for motif search (Fig. 2a)^34–36^. A total of 48 differentially expressed genes showed at least one CTCFL BSs in its promoter (Supplementary Data 2). Next, these genes were further analyzed by selecting the ones that show occupancy of CTCFL and CTCF using publicly available ChIP-seq data from Pugacheva et al.^11^, where six genes (Fig. 2b) showed ChIP-seq peak that matches the BSs identified (*VEGFA*, *IL12A*, *WSCD2*, *RBM20*, *TGFB1*, and *MAP1B*). Notably, *the CTCFL* gene contains a CTCFL BSs in its own promoter, in line with previous reports showing a CTCF-controlled transcription^37^. Furthermore, we also identified *TGFB1*, which was previously validated as a direct target of CTCFL^31^. Together, these results support our analyses and allow us to propose new previously uncharacterized CTCFL targets. In this regard, *VEGFA* is a previously uncharacterized target of CTCFL that plays a key role in angiogenesis^38^. We found *VEGFA* differentially expressed and its promoter is occupied by both CTCFL and CTCF in OC cell lines; thus, it is a potential direct target of CTCFL in OC. The latter is of great clinical relevance given the availability of precision therapies using VEGFA pathways as targets in other cancer types^39^ and growing evidence shows that this could be a promising target in OC^40^.

Next, in order to identify an underlying relevant protein pathway affected by the presence of CTCFL we obtained the largest interaction network enriched with the DEGs using KeyPathwayMiner^41^, allowing one exception (which is relevant to build the network and might have a mechanistic relevance) (Fig. 2c). The resulting network thus contains 32 CTCFL-driven DEGs and one exception. Functional evaluation genes in the network show that they participate primarily in the PI3k-Akt signaling pathway (*FGFR1, ITGB3, ITGB6, FN1, VEGFA, NTF3*, *and IL7R*) and are also involved in an extracellular matrix organization (*NCAM1, ITGB3, ITGB6, FN1, COL26A1*, and *CAST*) (Fig. 2d).

Notably, regarding the PI3K-Akt pathway, the proteins identified correspond mainly to the cell membrane receptors (FGFR1, ITGB3, ITGB6, and IL7R) and their ligands (VEGFA). Additionally, when examining protein complexes in the CORUM database^42^, we found that well-characterized functional protein complexes are deregulated by CTCFL; such as the ITGA2B-ITGB3-FN1-TGM2 complex (CORUM 2376) known to participate in cell adhesion^43^, where three out of the four members of the complex are affected; and the ITGB6-FYN-FN1 complex (CORUM 2351), which is involved in focal adhesion^44^. The above is of great importance in OC since established evidence shows that this pathway is significantly deregulated in OC^5,45–47^. Notably, FN1 seems to play a special role in the downstream effects of CTCFL deregulation through the alteration of signaling pathways (PI3K-Akt), as a key component of the extracellular matrix and through their interaction with VEGFA and TGFB1, which are not only differentially expressed but also have CTCFL and CTCF occupancy in their promoter.

Together, this data provides more detailed insights into the cellular effects occurring due to the transcriptional changes driven by CTCFL. In particular, these results indicate that CTCFL could be an important factor driving changes in the extracellular matrix components, which in turn, lead to changes in cell migration. Furthermore, our data suggest that the PI3K-Akt signaling pathway might play an important role in OC patients with a high expression of *CTCFL*. Finally, FN1 appears to be one of the main effectors of CTCFL-driven downstream changes in OC tumor cells.

### CTCFL-induced protein interaction network is deregulated and associated with survival in OC

Following the identification of a relevant interaction network enriched with DEGs due to the presence of CTCFL (33 genes), we aimed to evaluate whether these play a relevant role in OC patients, either by being associated with the survival and progression prognosis or by being druggable targets for treatment.

**Fig. 3:**
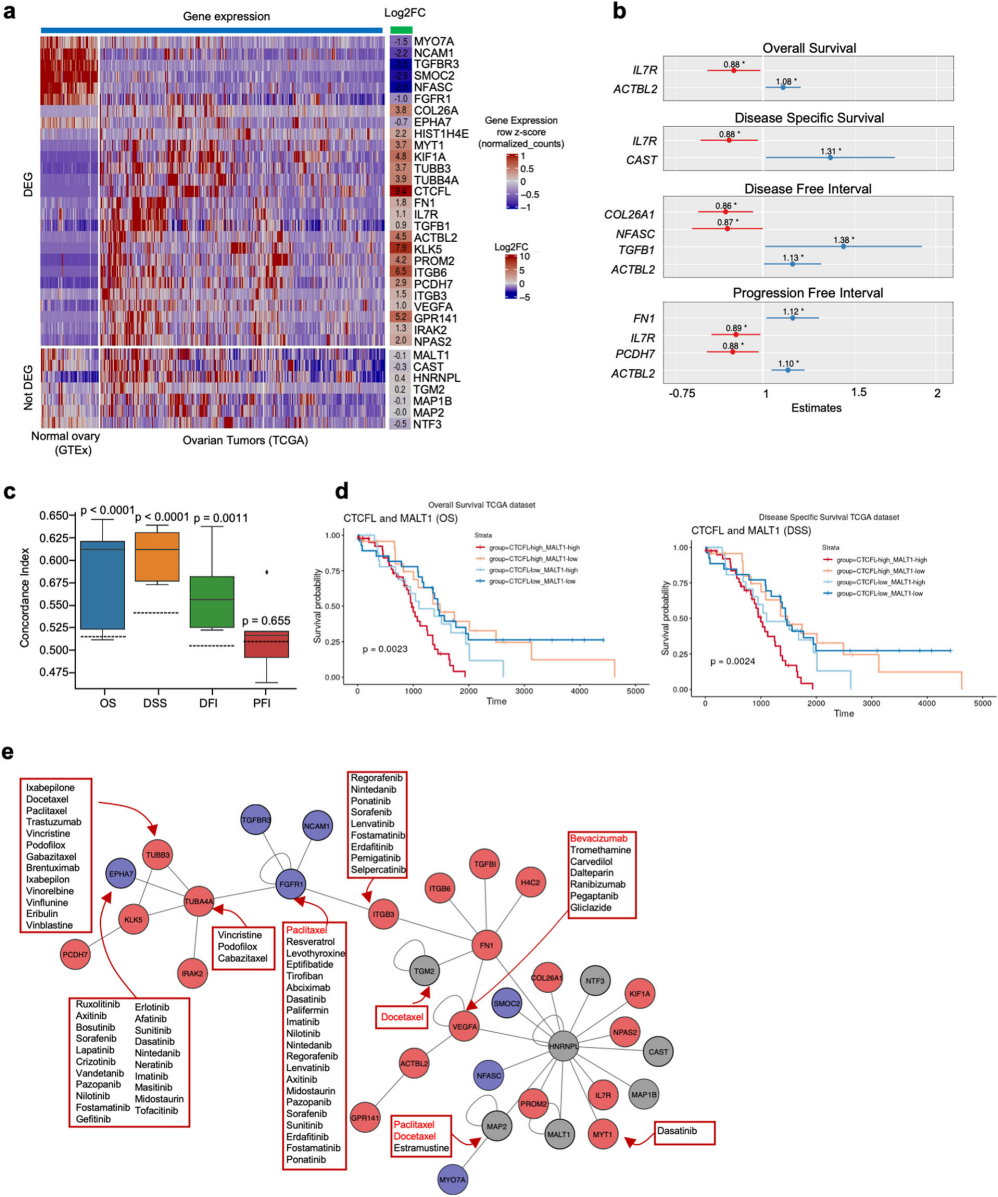
Evaluation of prognosis and druggability of CTCFL-regulated genes in ovarian cancer tumor samples. **a** Relative expression of the 34 CTCFL-regulated candidate genes in normal and tumor samples (left panel) and their corresponding fold change (right panel). Genes are grouped into DEGs (*p*-adj < 0.05) or not DEGs (*p*-adj ≥ 0.05). **b** Survival analysis with Cox PH model using four survival data: overall survival (OS), disease-specific survival (DSS), and progression-free survival (PFI). **c** Average Concordance Index (CI) obtained with the gene expression of the 34 candidate genes using the random survival forest (RSF) model. The dashed horizontal line indicates the average CI of random sets of genes for each survival dataset. **d** Kaplan–Meier plots of *MALT1* (top relevant gene found by OS and DSS RSF models) in combination with *CTCFL* expression. **e** *CTCFL*-regulated protein interaction network found in vitro depicting the druggable targets that are differentially expressed in tumor samples versus normal tissue (TCGA vs GTEx). Red and blue nodes indicate genes upregulated and downregulated in tumor vs normal, respectively. Targeting drugs are shown in red boxes, red drug labels indicate drugs currently clinically used for OC treatment.

To address this question, we evaluated the expression profile of the 34 genes (33 previously found plus *CTCFL*) in the TCGA (Serous Cystadenocarcinoma) and GTEX (Normal Tissue) datasets. We found that 27 out of the 34 genes are differentially expressed (Fig. 3a) between tumor and normal tissue, including *VEGFA* and *IL7R*, and being *CTCFL*, *KLK5*, and *ITGB6* the most upregulated and *TGFBR3* the top downregulated genes (Supplementary Fig. 4a). Furthermore, genes that belonged to the ITGB6-FYN-FN1 and ITGA2B-ITGB3-FN1-TGM2 complex (*ITGB3, ITGB6*, *and FN1*) are also differentially expressed; as well as PI3K-Akt pathway genes (*FGFR1*). This indicates that the genes deregulated by CTCFL are not only involved in relevant cancer-associated processes (such as signaling pathways and extracellular component organization as described above), but they are also differentially expressed in OC patients.

After comparing the expression profile of the selected genes in tumor versus normal samples, we aimed to evaluate whether they have prognostic potential by doing a Cox proportional hazards (CPH) regression analysis and a Random Survival Forest (RSF) analysis. We evaluated four different survival data types: overall survival (OS), disease-specific survival (DSS), disease-free interval (DFI), and progression-free interval (PFI).

The CPH analysis (Fig. 3b) shows that 8 genes are associated with the survival of patients; particularly, *IL7R*, *PCDH7*, *COL26A*, and *NFASC* are associated with a protective effect (low expression is associated with poor prognosis), whereas *TGFB1, ACTBL2, CAST*, and *FN1* are associated with an increased risk (high expression is associated with poor prognosis). Also, we evaluated the potential to predict survival with RSF, a nonparametric ensemble method for the prediction of survival with the advantage that it does not depend on model assumptions^48^. We trained and further applied the model to the four datasets using the 34 candidate genes as features and we also evaluated randomly selected genesets of the same size. We found that the candidate set of genes found by our previous network analysis is capable of predicting the survival of patients with significantly higher accuracy (Concordance Index, CI) than the random genes for OS, DSS and DFI datasets (Fig. 3c). The feature importance (contribution of each gene to the prediction of survival) for each of these models shows that the top predictor gene is *MALT1* for OS and DSS, *PCDH7* for PFI, and *ACTBL2* for DFI (Supplementary Fig. 4b). Notably, *MALT1* was not previously found by the CPH analysis, and Kaplan–Meier plots also show the relevance of *MALT1* in combination with *CTCFL* expression in the survival of OC patients (Fig. 3d). Together, this suggests that the selected genes are not only CTCFL-driven genes involved in cancer pathways and deregulated in OC patients; but they are also significantly associated with the survival of these patients.

Once we addressed the relevance of candidate genes in the prognosis of OC patients, we finally evaluated whether these genes are potential druggable targets. We used the CoVex platform^49,50^ to obtain drugs targeting the genes in the DEG-enriched network (Fig. 3e). We found three drugs, out of the 15 total approved drugs for OC (Altretamine, Gemcitabine, Carboplatin, Topotecan, Thiotepa, Niraparib, Paclitaxel, Cisplatin, Olaparib, Hydroxyurea, Trabectedin, Melphalan, Bevacizumab, Doxorubicin, and Rucaparib); such as the first-line chemotherapy drugs Paclitaxel and Docetaxel and the antiangiogenic drug Bevacizumab. The most identified drugs are tyrosine kinase inhibitors (TKI), which target mainly EPHA7, FGFR1, and ITGB3 (Fig. 3d). The latter is of clinical relevance since ITGB3 appears to be a key connector gene in the CTCFL-DEG-enriched network, it participates in the PI3K-Akt pathway and it is differentially expressed in OC patients. Despite the importance as a prognostic marker of *MALT1*, no approved targeting drugs were found; thus, its therapeutic potential remains to be explored and it could be a good candidate for drug development. Together, these results provide mechanistic insights into ovarian cancer-associated transcriptional changes while also being potential markers of survival and targets for precision medicine therapies; in particular, in patients with *CTCFL* overexpression.

## Discussion

*CTCFL* has been widely reported overexpressed in several cancer types^16,17,51^ and proposed as a promising therapeutic target given its patterns of expression in human tissues. Previous research suggests that the oncogenic functions of CTCFL are due to the promotion of a stemness state^17,52,53^ by regulating several processes, including apoptosis^23,54–56^ and epithelial to mesenchymal transition (EMT)^52^. In ovarian cancer patients, *CTCFL* is overexpressed specifically in HGSC patients^22^ and it is associated with poor prognosis^24^. However, little is known about the molecular mechanisms that are driven by CTCFL expression. As the lack of therapeutic alternatives and prognostic biomarkers still is a massive problem in OC, the identification of such CTCFL-driven mechanisms can contribute to the development of systems medicine approaches and more complex prognostic biomarkers in OC. Here we show that the underexpression and overexpression of CTCFL produce changes in cellular processes related mainly to the PI3K-Akt signaling pathway and extracellular matrix-related genes. Furthermore, these genes, together with *CTCFL*, are good mechanistic predictors of survival and can also be targeted by drugs currently being used or tested in other cancer types.

The PI3K-Akt pathway is the second most frequently altered pathway after p53^5^ and is proposed as a useful approach for therapeutic intervention in OC^57^, given its association with chemotherapy resistance^58–60^ and poor prognosis in OC^61^. We found several membrane receptors and their ligands, members of the PI3K-Akt pathway, as the key deregulated genes by CTCFL; most notably, EGFR1, ITGB3, ITGB6, and FN1 are deregulated by CTCFL in vitro and are also deregulated in OC patients. EGFR, ITGB3, and ITGB6 are membrane receptors that have AKT as a downstream target, and thus the inhibition of these could be a useful approach for OC therapy in patients showing chemotherapy resistance. Importantly, to our knowledge, no PI3K-Akt targeting drugs have been approved for the treatment of OC to date. However, preclinical evidence shows that the inhibition of EGFR-AKT signaling affects OC cell growth^62,63^. Further studies have been carried out to evaluate the performance of molecular inhibitors targeting PI3K or AKT in preclinical models^46,64^ and some inhibitors are downstream mTOR blockers with varying effectiveness^65^. Thus, the use of the RTK inhibitors (the upstream initiators of the PI3K-Akt pathway) to target the CTCFL-deregulated genes; such as EGFR1, could aid in improving the effectiveness of OC therapy. Furthermore, we found VEGFA, a key regulator of angiogenesis, deregulated by CTCFL in vitro and affected in OC patients. Targeting VEGFA in OC has been widely attempted with antibody-based therapies, such as Bevacizumab, which is FDA approved for the treatment of resistant OC^66,67^; however little therapeutic benefit has been observed^68^. Thus, the use of antiangiogenic therapies or anti-PI3K-Akt signaling pathways, in patients with overexpression of *CTCFL* or in combination with anti-CTCFL immunotherapy could be a promising therapeutic approach for drug-resistant tumors, since monotherapy in solid tumors appears to be ineffective^69^.

In addition to the described above, we identified several membrane transporters members of the Solute Carrier Family (SLC) and the ATP-binding cassette (ABC) differentially expressed in *CTCFL*-deregulated cells. SLC and ABC genes have been widely described as key mechanisms of resistance to Paclitaxel and Cisplatin, the current first chemotherapy option for OC patients^29^. We identified more than 50 members of the SLC and ABC family of transporters deregulated by CTCFL, thus suggesting that OC patients with deregulated levels of CTCFL protein could be associated with either resistance or sensitivity to chemotherapy. Previous studies in a neuroblastoma model indicate that the overexpression of *CTCFL* is related to a resistant phenotype, through the regulation of epigenetic processes^70^ and through the alteration of the ALK gene (whose downstream signal is the PI3K-Akt pathway as well)^71^. Also, previous studies suggest a role of CTCFL in drug resistance through the maintenance of a stemness state^17^. The latter supports our results that indicate that CTCFL can, not only serve as a therapeutic target but also could be associated with tumor chemoresistance in OC. However, further analyses are required in OC to validate the potential use of CTCFL as a predictor of chemoresistance.

In addition to the potential role as a therapeutic target, we found that CTCFL expression levels, in combination with the expression of other identified genes in our protein interaction network, provide a good prediction of survival. Notably, in patients with high expression of CTCFL, low levels of MALT1 indicate a good prognosis (OS and DSS). Previous studies report that MALT1 is a key regulator of chemoresistance in other cancer types^72^. This supports the idea that *MALT1* may be utilized as a biomarker of chemoresistance in combination with *CTCFL*.

As our analysis is mainly based on gene expression data, we cannot be fully certain that our marker genes’ expression is reflected in the abundance of the corresponding proteins. However, our results, together with the fact that CTCFL itself is a cancer biomarker and a target for immunotherapy^26^, suggest that the combinatorial use of CTCFL and its target genes have high utility as mechanistic predictors for survival and metastasis.

Taken together, our study elucidates the molecular mechanisms driven by the oncogene CTCFL in ovarian cancer, which may further be utilized as prognostic biomarkers as well as for targeted therapy and drug development. In particular, we found PI3K-Akt initiators; such as the RTKs EGFR1 and VEGFA, and the integrins ITGB3 and ITGB6, to be potential drug targets in ovarian cancer patients with high *CTCFL* expression. We also identified the additional CTCFL targets ACTBL2, MALT1, and PCDH7 to be predictive for ovarian cancer treatment outcomes.

## Methods

### Cell lines

The human epithelial ovarian cancer cell line OVCAR3 (ATCC HTB-161) was obtained from ATCC (Manassas, VA, USA) and subcultured in a complete growth medium contained RPMI-1640 Medium (Catalog No. 30-2001; ATCC), supplemented with 20% fetal bovine serum (Catalog No. 10437028; Gibco) and 0.01 mg/ml bovine insulin (Catalog No. I0516-5ML; Sigma). OVCAR3 cells were subcultured at 37 °C in a 5% CO_2_ incubator.

### Cell transfections

The plasmid pCpGfree-vitroHBORIS contains *CTCFL* ORF encoding the canonic protein of 663 amino acids. pCpGfree-vitroHLacZ plasmid was used as a negative control. Both constructs were kindly donated by Prof. Lobanenkov and Dr. Pugacheva^11^. pCpGfree-vitroHBORIS and pCpGfree-vitroHLacZ (Catalog No.cat. no. pcpgvth-lz; InvivoGen) plasmids were cloned in *E. coli* GT115 (Catalog No. gt115-11; InvivoGen) and Hygromycin B (Catalog No. 10 843 555 001; Sigma) was used as selection antibiotic. Plasmid purification was performed with QIAEX II Gel Extraction Kit (Catalog No. 20021: QIAGEN). OVCAR3 cells grown to 70% density were transiently transfected with pCpGfree-vitroHBORIS (CTCFL-OE) or pCpGfree-vitroHLacZ (Control) plasmids, using Xfect Transfection Reagent (Catalog No. 631317; Clontech) and 2 µg of plasmid, transfections were performed overnight. Cells were collected 48 h post-transfection for RNA and protein extraction. Three technical replicates were pooled together to obtain one biological replicate. Three independent biological replicates were performed.

### Protein extraction and western blot

Collected cells were washed with ice-cold PBS solution and resuspended in RIPA buffer with protease inhibitor cocktail cOmplete (Catalog No. 4693132001; Roche). Protein quantification was performed with DC protein assay (Catalog No. 500-0116; Bio-Rad) and the signal was obtained with iMark™ Microplate Absorbance Reader (Catalog No. 1681130; Bio-Rad). Next, 10 µg of total protein per sample was prepared for electrophoresis in 10% bis-acrylamide SDS-PAGE gel. Proteins were transferred to a PVDF membrane overnight. Antibodies used for immunodetection are Anti-CTCFL (Catalog No. HPA001472; SIGMA Aldrich) dilution 1;10, Anti-GAPDH (Catalog No. SC-25778; Santa Cruz Bt.) dilution 1:2500, and HRP anti-rabbit (Catalog No. SC-2004; Santa Cruz Bt) dilution 1:5000. CTCFL abundance was determined by standard densitometry analysis using ImageJ software (NIH, USA) (Fig. 1a and Supplementary Fig. 1a). Signal normalization was performed using GAPDH (Catalog No. SC-47724; Santa Cruz Bt). Gels are derived from the same experiment and were processed in parallel.

### RNA extraction and RNA-sequencing

Total RNA from CTCFL-OE and Controls was extracted using TRIzol Reagent (Catalog No. 15596026; Invitrogen) and Direct-zol RNA Miniprep (Catalog No. R2051; Zymo). RNA from CTCFL-KD was obtained from Knockdown experiments validated and published previously^7^. The integrity and quality of RNA was analyzed with TapeStation 2200 (Agilent, USA). Sequencing was performed by Unidad de Secuenciación (INMEGEN, Mexico City). mRNA for NGS sequencing was quantified with Qubit 4 (Thermo Fisher Scientific, USA) and libraries were prepared with TruSeq Stranded mRNA Library Prep (Illumina). Sequencing was performed in a NextSeq 500 platform (Illumina) with 25 M reads per sample, paired-end, and 75 bp read length.

### Differential expression analysis

Initial quality assessment and trimming of low-quality reads was performed with FastQC v0.10.1^73^ and Trimmomatic v0.27^74^. Alignment to the genome was performed using STAR^75^ and the Homo sapiens reference transcriptome (GRCh38). Counts were obtained with FeatureCounts from the R package Rsubread v1.32.4^76^. Differential expression analysis was performed with DESeq2 v1.22.2^77^, genes were selected with |Fold Change | > 2 and FDR *p*-adj < 0.1 (for CTCFL-OE vs Control) and FDR *p*-adj < 0.05 (for CTCFL-KD vs Control). Differentially expressed genes are shown in Supplementary Data 1. Differentially expressed genes identified in each comparison were used for Functional Enrichment Analysis with gProfiler a cutoff of *p*-adj < 0.001 (for CTCFL-OE vs Control) and *p*-adj < 0.05 (for CTCFL-KD vs Control).

### CTCFL binding site identification

Sequences flanking the TSS (2 Kbp) of DEGs identified in CTCFL-OE and CTCFL-KD were obtained using Biomart^78^. HMMER 3.2.1 software^36^ was used to search for CTCFL BSs using the known motifs reported in Jaspar database^34^ (profile MA1102.1) and Factorbook^35^. Clustal Omega software^79^ was used for the alignment of known motifs. For regions with more than one identified BS, only the site with the best e-value was selected. Two independent searches with HMMER were performed, one for each CTCFL motif (Jaspar and Factorbook). Genes with identified BS by both independent searches and position in the same genomic coordinate were selected. (Supplementary Data 2).

### ChIP-seq data integration

WIG formatted ChIP-seq data for CTCFL and CTCF in OVCAR8 cell lines was obtained from Pugacheva et al.^11^ available in GEO database (GEO accession GSE70764)^80^. Coordinates were converted to GRCh38/hg38 genome assembly using the liftOver tool from UCSC Genome Browser^81^. Visualization of ChIP-seq peaks was performed with IGV software^82^.

### Network analysis and target-drug associations

Differentially expressed genes identified in CTCFL-OE and CTCFL-KD were used as active genes to create a custom indicator matrix as input for *de novo* pathway enrichment with KeyPathwayMiner^83^ using a physical interaction network obtained from BioGrid and the parameters K = 1, L = 0. Cytoscape was used for network visualization^84^.

To retrieve drugs targeting proteins in the network, gene names were mapped to UniprotIDs and input into CoVex^49,50^; followed by the MuST algorithm with default parameters (except including non-seed viral proteins=IGNORE) and the Closeness centrality algorithm with default parameters and result size = 500.

### Expression analysis in tumor and normal ovarian samples

For differential expression analysis of tumor *vs* normal ovarian samples, RSEM_expected counts were downloaded from the TCGA, TARGET, and GTEx dataset from the UCSC Toil RNA-seq Recompute^85^ available through the Xena Browser^86^. A total of 88 normal (GTEx) and 419 neoplastic (TCGA) ovarian samples were retained after removing recurrent tumor samples. Counts were subjected to differential expression analysis using DEseq2^77^. Differentially expressed genes were selected with abs(Fold Change) > 1.5 and *p-*adj < 0.05.

For survival analysis, gene expression (RSEM tpm) of the TCGA, TARGET, and GTEx datasets was downloaded from the UCSC Toil RNA-seq Recompute^85^. A total of 419 neoplastic (TCGA) ovarian samples were retained after removing recurrent tumor samples. Four different outcome labels were used, producing four separate datasets: overall survival (OS, 417 samples), disease-specific survival (DSS, 387 samples), disease-free interval (DFI, 202 samples), and progression-free interval (PFI, 417 samples).

Preprocessing, Kaplan–Meier estimation, Cox proportional hazard model (CPH), and random survival forest (RSF) were applied to all datasets. For the preprocessing, the datasets were checked for high-correlating features (Pearson correlation coefficient >95%), where zero genes showed high correlation, thus all genes were retained. For Kaplan–Meier estimation and CPH Model, the lifelines package^87^ was used; in particular, for CPH, a five-times repeated fivefold cross-validation was used and the concordance index (c-index) was used as an evaluation metric. RSF analysis was performed with the scikit-survival package^88^ by splitting the dataset into 80% train and 20% test. On the training set, a randomized hyperparameter search was performed with a five-times repeated fivefold cross-validation and evaluated using the c-index. The hyperparameters of the best-performing model of the randomized search were used to train the actual model on the whole training set, which was evaluated on the 20% unseen test data. Finally, the feature importance of the model was computed.

## Supplementary information

Supplementary Figures

Supplementary Data 1

Supplementary Data 2

## Data Availability

The datasets generated and analysed during the current study are available in the GEO repository, GSE166767.
