# Supplementary material for: CTCFL regulates the PI3K-Akt pathway and it is a target for personalized ovarian cancer therapy
Source: NPJ Syst Biol Appl. 2022 Feb 7;8:5. doi: 10.1038/s41540-022-00214-z (PMC8821627; doi:10.1038/s41540-022-00214-z)
Supplement: Supplementary file 1 — Supplementary Figures [file 41540_2022_214_MOESM1_ESM.pdf]

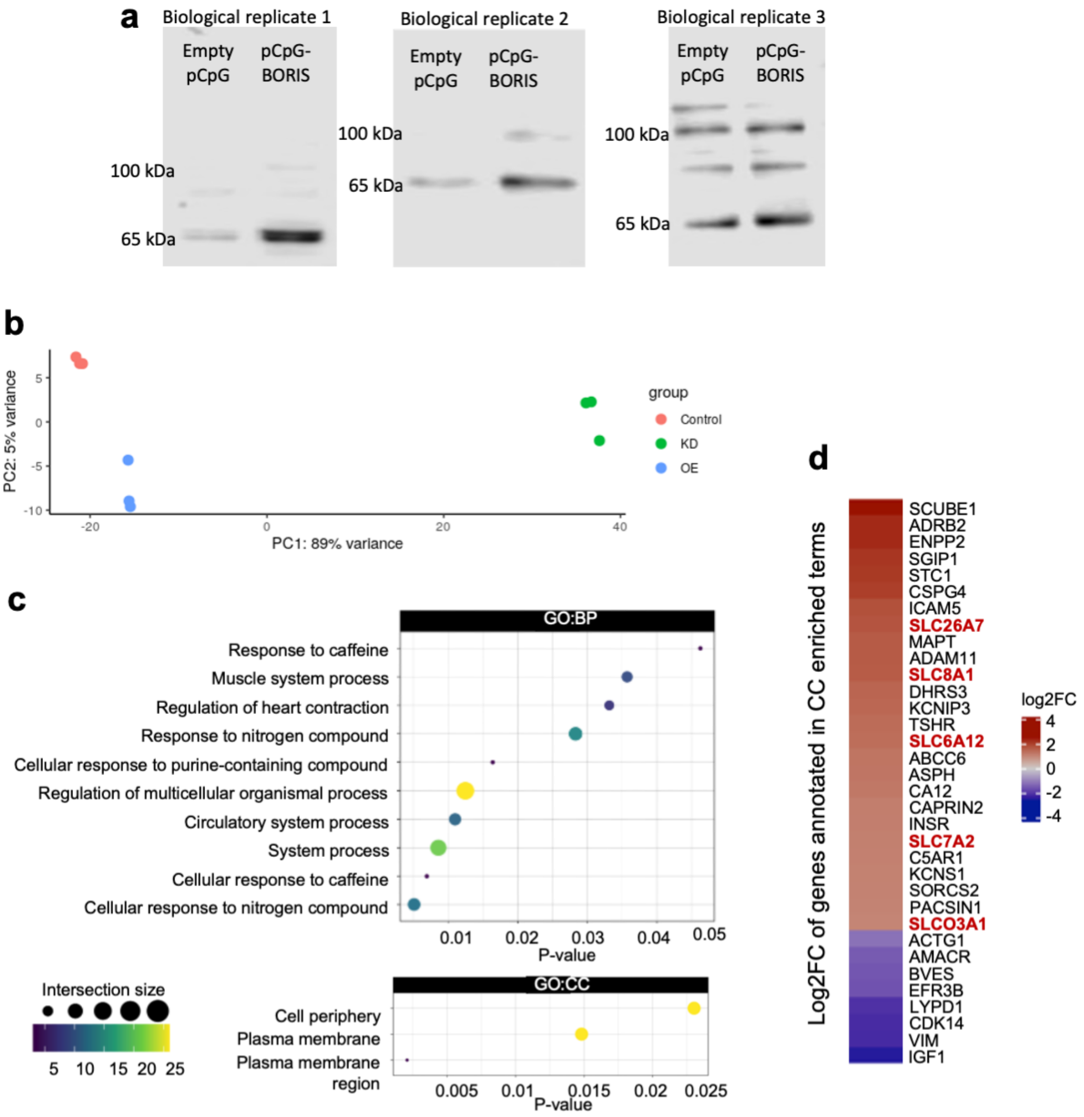

Supplementary Figure 1. Functional analysis of DEGs in CTCFL-OE (86 genes). a) full un-cropped image of CTCFL blots of each biological replicate. The canonic protein corresponds to the 65 kDa band. **b)** PCA depicting the three experimental conditions and their biological replicates. **c)** Gene Ontology enriched terms in Biological Process (BP) and Cellular Component (CC). No significant enriched terms were obtained in Molecular Function. Enriched terms were selected with padj < 0.05. **d)** Fold Change of genes contained in GO:CC terms Cell periphery, Plasma membrane and Plasma membrane region. Red labeled genes are members of the solute carrier family (SLC).

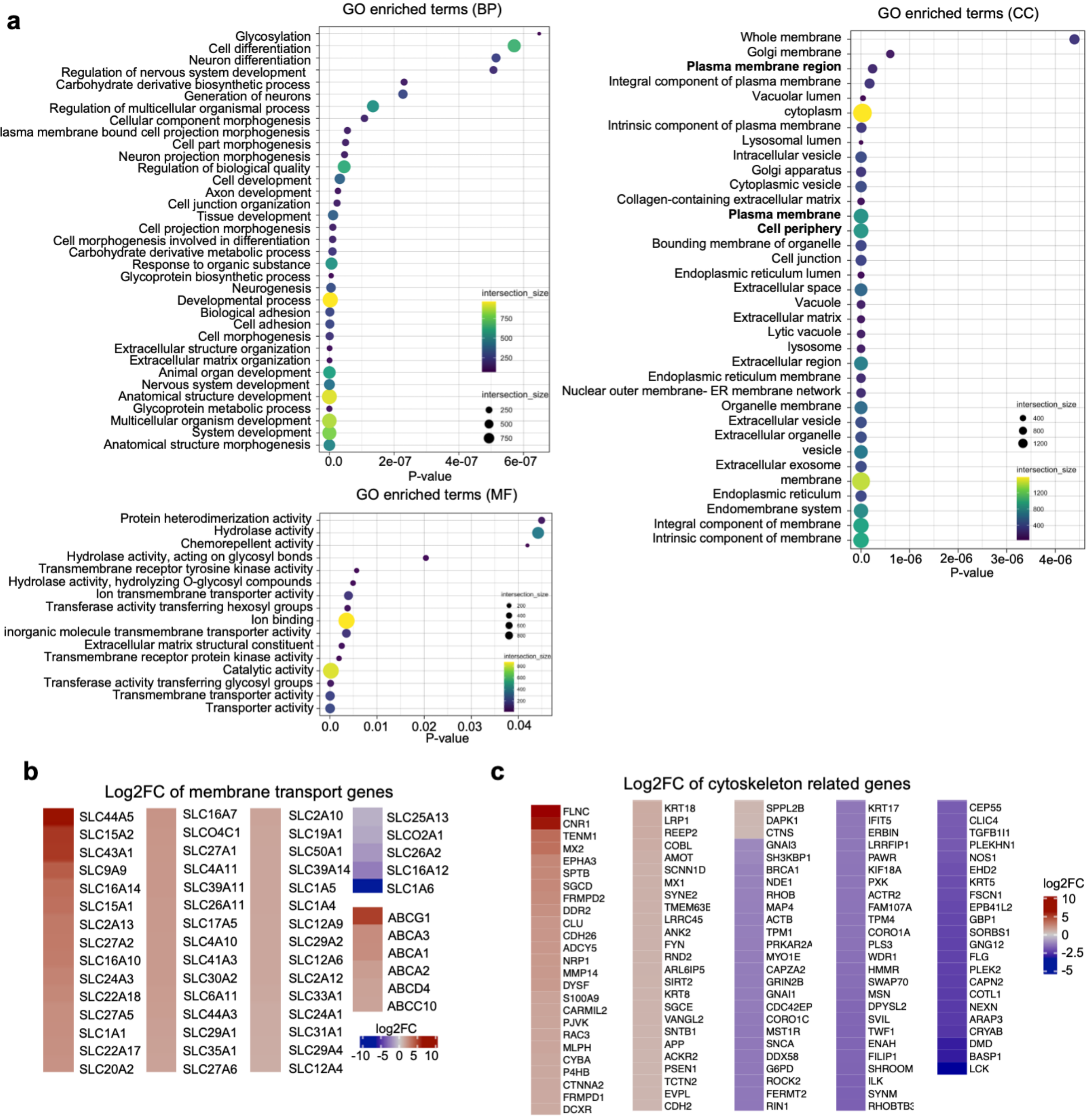

Supplementary Figure 2. Functional analysis of DEGs in CTCFL-KD (2713 genes). a) Top 35 Gene Ontology enriched terms in Biological Process (BP), Cellular Component (CC) and Molecular Function (MF). **b)** Fold Change of genes members of the Solute Carrier Family (SLC) or ATP-binding Cassette (ABC) transporter family which are differentially expressed in CTCFL-KD. **c)** Fold Change of genes annotated for Homo sapiens in Cytoskeleton GOterm (GO:0005856) which are also differentially expressed in CTCFL-KD.

a

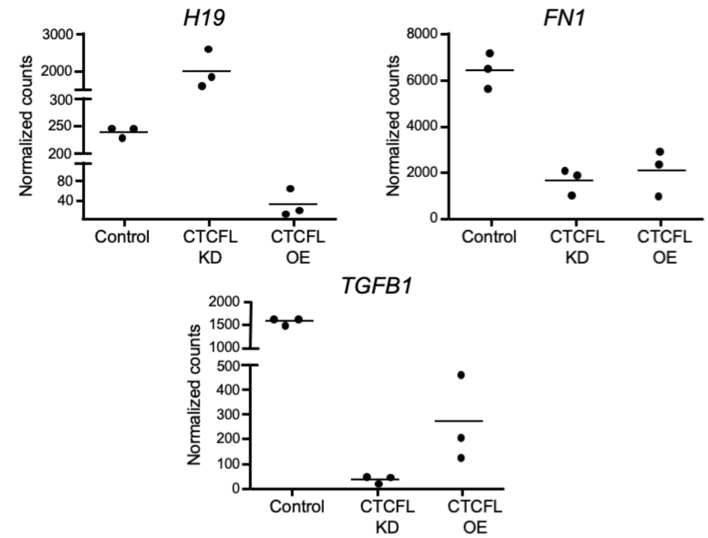

b

	CTCFL-OE		CTCFL-KD	
Gene	Log2FC	AdjP-value	Log2FC	AdjP-value
FN1	-1.59	0.039	-1.95	0.006
H19	-2.54	$3.96x10^{-5}$	3.01	$7.05x10^{-6}$
TGFB1	-2.49	$1.42x10^{-5}$	-5.49	$1.23x10^{-18}$

c

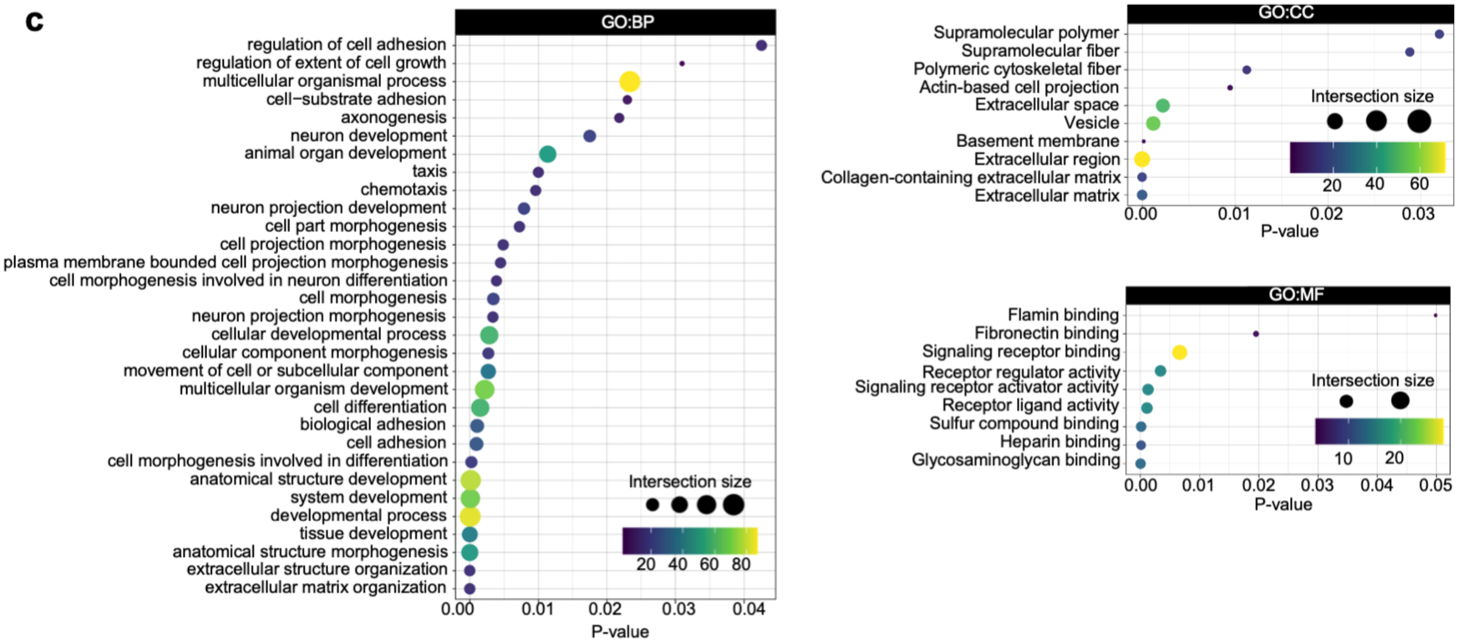

Supplementary Figure 3. Functional analysis of DEGs in both CTCFL-KD and CTCFL-OE (149 genes). a) Gene Ontology enriched terms in Biological Process (BP), Cellular Component (CC) and Molecular Function (MF). **b)** Transcript levels of previously reported CTCFL target genes (*H19, FN1* and *TGFB1*) in each experimental condition. **c)** Change in gene expression and statistical confidence of previously reported CTCFL target genes (*H19, FN1* and *TGFB1*) in each experimental condition vs control.

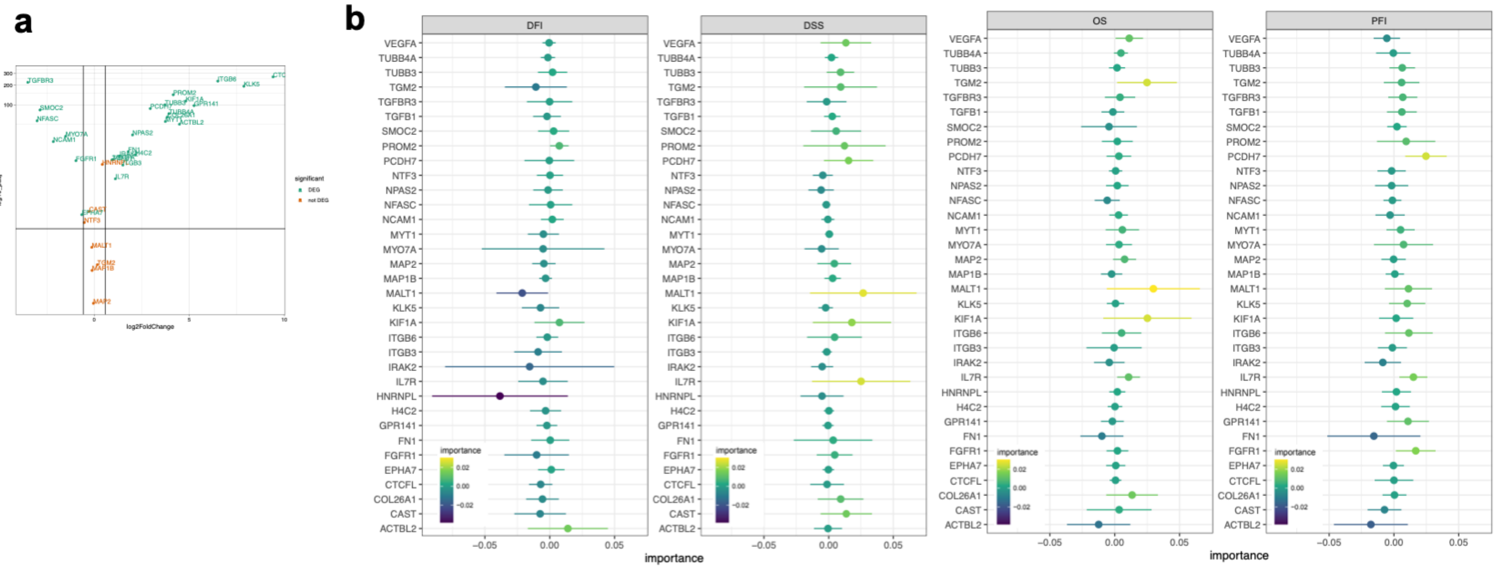

Supplementary Figure 4. Evaluation of CTCFL-regulated candidate genes in OC samples (TCGA). a) Volcano plot of the 34 CTCFL-regulated genes in tumor (TCGA) vs normal (GTEx) samples. **b)** Feature importance obtained with the Random Survival Forest analysis for each survival dataset.
